# Design, Implementation and Data Analysis of an Embedded System for Measuring Environmental Quantities †

**DOI:** 10.3390/s20082304

**Published:** 2020-04-17

**Authors:** Martin Pieš, Radovan Hájovský, Jan Velička

**Affiliations:** Department of Cybernetics and Biomedical Engineering, VŠB-Technical University of Ostrava, 17. listopadu 2172/15, 70800 Ostrava, Czech Republic; radovan.hajovsky@vsb.cz (R.H.); jan.velicka@vsb.cz (J.V.)

**Keywords:** carbon dioxide, IQRF^®^, NDIR, measurement network, wireless sensor network

## Abstract

The article describes the development and implementation of a complex monitoring system for measuring the concentration of carbon dioxide, ambient temperature, relative humidity and atmospheric pressure. The presented system was installed at two locations. The first was in the rooms at the Department of Cybernetics and Biomedical Engineering, Faculty of Electrical Engineering and Computer Science, VŠB-Technical University of Ostrava. The second was in the classrooms of the Grammar School and Secondary School of Electrical Engineering and Computer Science in Frenštát pod Radhoštěm. The article contains a detailed description of the entire measurement network, whose basic component was a device for measuring carbon dioxide concentration, temperature and relative humidity in ambient air and atmospheric pressure via wireless data transmission using IQRF^®^ technology. Measurements were conducted continuously for several months. The data were archived in a database. The article also describes the methods for processing the data with statistical analysis. Carbon dioxide concentration was selected for data analysis. Data were selected from at least two different rooms at each location. The processed results represent the time periods for the given carbon dioxide concentrations. The graphs display in percent how much of the time students or employees spent exposed to safe or dangerous concentrations of carbon dioxide. The collected data were used for the future improvement of air quality in the rooms.

## 1. Introduction

Improving the quality of our environment today is a relevant topic, especially regarding global warming and the occurrence of greenhouse gases. One of the areas of focus which has a significant impact on human health is the quality of work environments. These issues are linked to work hygiene and the types of environment people work in. In recent years, carbon dioxide concentration has started to be very closely monitored, as increased concentrations of this gas can result in impaired labor productivity. These issues are also very applicable to educational environments. In this regard, students’ concentration and attentiveness in classrooms are a significant factor.

Carbon dioxide concentration influences overall air quality. In interior rooms, CO_2_ concentration should be less than 1000ppm. Exceeding this concentration adversely affects mental performance and concentration, and with elevated levels, can trigger headaches and fatigue. If a concentration of 5000ppm is exceeded, severe health risks are imminent.

In school environments, values for temperature and humidity are essential. These are stipulated in Annex 3 to the Directive of the Ministry of Education, Youth and Sports No. 343/2009 (on children and juveniles). The values for school classrooms and office rooms in this directive were selected for this study [1].

The Directive of the Ministry of Regional Development No. 20/2012 (on technical requirements for buildings) stipulates a maximum permissible CO_2_ concentration of 1500ppm in rooms occupied by people. Values above this limit can be defined as unsuitable. The limit values for the measured quantities in this study were therefore specified according to these regulations (Table 1).

This paper describes the device first introduced in [2,3] and used to measure ambient carbon dioxide concentration, temperature and relative humidity and atmospheric pressure. The paper [2] very briefly describes the measurement chain for monitoring carbon dioxide in the department where the authors work. There is no detailed description of the measurement chain and no more detailed statistical evaluation of the data. The article [3] describes the first-generation device for measuring CO_2_ concentration, temperature and relative humidity of ambient air and atmospheric pressure. At the end of the article, there is shown the practical output of measured quantities and their statistical processing.

Monitoring carbon dioxide concentration at the Department of Cybernetics and Biomedical Engineering was based on the thesis presented in [4]. Ref. [4] refers to the diploma thesis of Mr. Jan Velička. The supervisor was Ing. Martin Pieš, Ph.D. This work is a detailed description of the development of the first-generation service for measuring the CO_2_ concentration, temperature and relative humidity of ambient air and atmospheric pressure, including production materials. This thesis is in the Czech language. At that time, monitoring carbon dioxide concentration in these areas due to people’s levels of fatigue became necessary. Article [5] describes the effect of the dependence of carbon dioxide concentration on the number of persons in a monitored environment. 20-day monitoring of one university room was performed and the measured data were compared in graphs depending on the number of persons. For their experimental measurements, the authors of the article used an e-nose for indoor odor monitoring and a photoacoustic multi-gas instrument for carbon dioxide detection.

Article [6] describes an interesting solution for monitoring CO_2_ concentration. The method used a sensor hidden in a table lamp. This so-called smart desk lamp monitors the carbon dioxide concentration in its vicinity and triggers room ventilation using measured and analyzed data. XBee technology was used to transmit the measured carbon dioxide concentration data.

This paper describes the measurement of environmental quantities in the laboratories of the authors’ department and in the Grammar School and Secondary School of Electrical Engineering and Computer Science, Frenštát pod Radhoštěm. For this purpose, a Room Measurement of Carbon Dioxide (RMCD) device was developed using IQRF^®^ technology described in [7,8] for transmitting measured environmental data. For the transmission of information from the measuring nodes, we decided to use wireless technology, which allows bi-directional communication in the MESH topology due to possible extension of the monitoring system with control elements. IQRF^®^ technology seems to be a suitable adept, which allows creating large wireless sensor networks with bi-directional communication among their nodes in MESH topology. Nodes can communicate at any time, even in the mode of reduced power consumption. Another reason for choosing this technology is the authors’ long experience in the implementation and use of this technology in the field of wireless sensor development, which is supported by several successfully solved R&D projects. Reference [7] is a website of the manufacturer of IQRF^®^ technology, where it is possible to download complete documentation for this technology. The article [8] describes the connection of IQRF^®^ technology to the Raspberry Pi microcomputer. There are described individual modes of reduced consumption of IQRF^®^ modules and the principle of communication in the IQRF^®^ network. This article is an older date and does not cover the latest advances in communication protocol in IQRF^®^ networks.

This paper extends the original article [9] on the statistical analysis of measured data at the Department of Cybernetics and Biomedical Engineering at VŠB-TUO and also describes the installation of a measurement network at the Grammar School and Secondary School of Electrical Engineering and Computer Science, Frenštát pod Radhoštěm. These two sets of measurements form two individual case studies. The first case study describes the installation of 8 Room Measurement of Carbon Dioxide (RMCD) devices where only CO_2_ concentration and ambient air temperature were measured. The second case study describes the installation of 43 RMCDs, which, in addition to the first two quantities, also monitor the relative humidity of the ambient air and atmospheric pressure. Atmospheric pressure data are not analyzed in this paper. It is for informational purposes only.

The case studies described in this paper contain a description of a measurement network that uses a commercial IQRF^®^/Ethernet gateway with the connection to IQMESH measurement node network with wireless transmission to an IQRF^®^ network. The data processing of the measured data is done at the university server.

### Related Work

Several types of sensors are commercially available for measuring CO_2_ concentration. Usually, they are simpler sensors indicating the measured level of CO_2_ concentration by means of a color LED [10]. Some sensors also allow relative humidity measurements [11]. These sensors are powered by alkaline batteries and/or by an AC adapter. There are also sensors that indicate the level of the measured CO_2_ concentration on the display. Some sensors have a relay output to switch on, e.g., ventilation when the set CO_2_ concentration limit is reached. These simpler sensors have no connectivity to wireless networks. The price of these sensors ranges from 100 EUR to 360 EUR. An example of such a sensor can be seen here [12]. The use of this type of sensor in the form of a data logger is described in [13]. In this paper, however, the analysis of data is only marginally addressed.

More complex and expensive sensors already allow wireless communication, e.g., with a mobile phone via Bluetooth or Wi-Fi. However, this wireless communication is only peer to peer. The advantage is the possibility to display the current measured values on the display of a mobile phone or tablet. It is also possible to view historical trends and, in some cases, to set an event that occurs when the measured value is exceeded. The disadvantage is a higher power consumption and therefore less battery life. Some types of sensors have a solution in that they use rechargeable LiPol batteries, for example, which are recharged via the AC adapter. Sensor communication via the IEEE 802.15.4 standard is described in [14]. This article uses sensor communication in MESH topology, specifically a RapidMesh OPM15 board is used as the radio module. However, the reference in the cited article to the manufacturer of this device is no longer available.

There are also commercially available sensors for e.g., the Z-Wave standard. For this technology, which is directly designed for building automation, there is a sensor [15] that measures CO_2_ concentration, temperature, and humidity. Unlike our wireless sensor, it cannot measure atmospheric pressure and battery operation is not possible. Wireless sensors are primarily intended for implementation in existing “unintelligent” buildings, especially schools which are not equipped by industry standards for building automation such as KNX [1]. That is why our environmental sensor has been designed as a wireless sensor that can be used to create an extensive wireless sensor network without any building modifications to existing buildings.

Sensors that have connectivity to IoT networks such as Sigfox or LoRa (collectively referred to as LPWAN) are also commercially available, see [16]. Most commercially available Sigfox-based systems are unsuitable for large wireless sensor networks due to the high cost of individual licenses [17,18]. Commercial solutions such as GlobalSat LS-111 [19], ref. [16] based on LoRaWAN technology are dependent on the infrastructure of the provider, which usually charges additional fees. For this reason, these solutions are not suitable for monitoring many rooms within a building. For example, ref. [20] sets a monthly fee of 1 EUR for a device that sends at least one message per day. However, this solution only includes 100 historical values and a web interface with basic charts. A more expensive option, 40 EUR per month includes 100 sensors, support 5 gateways, MQTT broker services, and REST-API, so it could be used as an alternative to our solution. However, as will be described below, the use of, for example, 40 commercially available sensors to monitor environmental quantities in a building is unnecessarily expensive to operate. These commercially available sensors communicate over distances in the range of units up to tens of kilometers and unnecessarily consume ISM bandwidth. Although LoRaWAN technology allows reducing the transmitting power of its wireless nodes, the use of star topology in LPWAN technologies does not effectively cover the entire building to ensure reliable communication. As mentioned above, we decided to use the MESH topology, which, even at low transmission power of individual communication nodes, allows reliable coverage of all places in the building without the need to disrupt the ISM band outside the monitored buildings. IQRF^®^ technology provides the ability to remotely adjust the transmit power. With lower transmission power, it allows the sending of data only within the monitored building. This creates a personal area network (PAN). Another advantage is that the sensors in IQMESH transmit bi-directionally with AES-128 encryption and there are no license fees.

Wireless sensor networks using a star topology for data transmission are not suitable for creating large, robust measurement systems that need to communicate in both directions. The main limitations include the need for direct node-gateway communication, limitations on the number of messages per day, or the cost per license for each node. For example, sensors using Sigfox technology can send 144 messages and receive only 4 messages per day. This significantly reduces the reconfiguration of sensor settings with Sigfox. LoRa allows the downlink channel but only to a limited extent, according to the division of LoRa modules into three classes, A, B, and C [21]. Class A devices only have two very short receive windows after they have transmitted a packet. After the receive windows, the class A device goes to sleep to conserve energy. In addition to class A device that has a receive windows, class B devices open extra receive windows at scheduled intervals. The receive windows are synchronized by beacons sent from the gateway. Last, class C devices, as they are usually not battery-powered, can afford to continuously have their radio in receive mode (as long as they are not transmitting themselves), allowing for instantaneous transmission of data towards a device without having to wait for a receive window to open [22]. IQRF^®^ technology allows bi-directional communication of 64 Bytes of data at 19.2 kbps in low-power mode, where the IQRF^®^ TR-76D transceiver itself consumes 250 μA in low-power mode. Sensors using Sigfox or LoRa technology are useful in places where it is necessary to monitor a few physical quantities of one building, such as reading water or gas meters, where the license fees for the operation of this monitoring system can be integrated into energy bills.

If we used Sigfox technology to transmit the measured environmental quantities in the classrooms, the period of 6 measurements per hour would be insufficient. Lessons are usually organized into 45-min blocks, which are interleaved with 10 to 30-min breaks. Therefore, it is advisable to increase the measurement period to the maximum possible. Even from the point of view of statistical processing of measured data, a measurement period of 6 samples per hour could have influence due to ventilation, which is usually shorter than the period without ventilation. Using LoRa technology we could create a personal area network (PAN) but at the cost of a significant implementation effort. LoRa modules do not natively support communication in MESH topology. The use of MESH topology in LoRa modules is discussed in [23]. However, it is only a pilot prototype solution that is programmed in the LoRa module and is not standardized in any way.

Another disadvantage of using a star topology in our application is that in this topology, each sensor communicates with the base station. This way of communication is very energy-intensive (e.g., Sigfox, LoRa technology). Wireless sensor networks with MESH topology have one network coordinator and are assigned individual wireless nodes (sensors). The energy requirements of each node depend on the distance from the coordinator and on how many neighboring nodes it forwards messages. Further in the text, see Section 2.1, there is an example of the discovered IQMESH network. Another immense advantage is the high robustness and reliability of communication [24].

Summarizing the shortcomings of commercially available wireless data sensors that communicate on a LPWAN, the following statements are made. LPWAN technologies based on higher communication range devices and star topology networks are generally characterized by high connection costs. Currently, it is possible to get one device connection for approx. 1 EUR per month (Sigfox) [18]. Insufficient transmission reliability for control purposes, the impossibility of OTA (Over The Air) updates reduces security (Sigfox), short packets with only 12 B of user data do not allow the implementation of the expected security and encryption standards. Long broadcasting of a single message also means communication restrictions on the number of messages sent due to legislative restrictions in most countries, which LPWAN technology handicaps in many application areas where a faster response from the system is needed. The security of the IQMESH wireless network itself is based on the AES-128 encryption standard. It also includes data fraud protection, data consistency checking, man in the middle (MITM) attack resistance, and a key management system. An important part of security is also the possibility to change or extend the security of network elements using OTA (firmware changes over the wireless network) [25,26]

For all the reasons described above, we have decided to develop our own sensor, which will allow the measurement of CO_2_ concentration and temperature, humidity and atmospheric pressure. We also decided to use IQRF^®^ technology for wireless communication. It allows the creation of large sensor networks based on MESH topology. This technology is ideal for the application of sensors, e.g., for measuring CO_2_ concentration in many classrooms, as described later in the article. Another advantage of our solution is the possibility of both battery and mains supply of the sensor. This ensures continuous sensor operation even in the event of a power failure. The device also allows direct visualization of the CO_2_ concentration and thus can operate autonomously without having to send data to the master system. This provides immediate feedback to users staying in the monitored room. Another advantage of the described solution is acoustic signaling when the limit concentration of CO_2_ is exceeded at 5000ppm. As already mentioned, another advantage is that IQRF^®^ technology enables bi-directional communication with our wireless sensors and communication is done with AES-128 encryption and no license fees are applied to individual sensors.

Our designed and constructed solution of the monitoring system for measuring environmental quantities is fully applicable to measurement requirements at several tens to hundreds of places simultaneously (classrooms, rooms, laboratories). Data from these locations are concentrated on one superior system, which ensures high robustness and speed of visualization of measured quantities.

## 2. Materials and Methods

### 2.1. Carbon Dioxide Measurement Device

**Figure 1:**
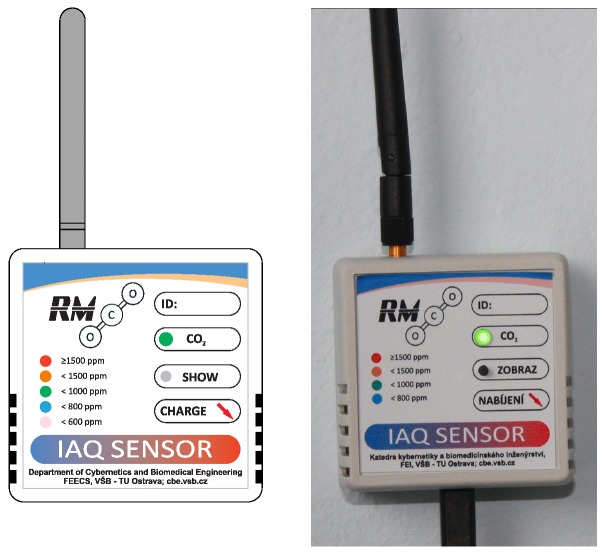
Diagram and photograph of installed RMCD device.

The proposed measurement network for monitoring air quality in the Department of Cybernetics and Biomedical Engineering laboratories was based on wireless measurement nodes called RMCDs (Room Measurement of Carbon Dioxide). These devices are permanently powered from the mains via a micro USB power adapter. By using this connector, powering this device from any commercially available power adapter with a 5V supply voltage and current of at least 500mA is possible. A photograph of one of the RMCD devices is shown in Figure 1.

**Figure 2:**
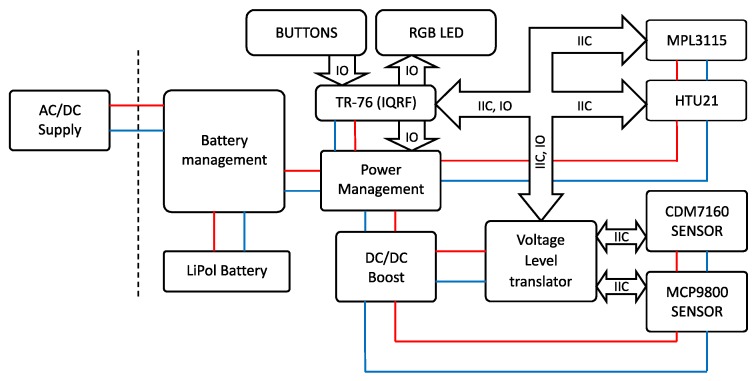
Internal structure of the RMCD device.

The internal structure of the RMCD is shown in Figure 2. This block diagram of the wireless measurement module shows the links between the blocks [4].

As already described, the module is powered from a USB power supply. Battery management handles recharging of a 3.7V/1100mAh Lithium Polymer battery as well as short-circuit and under-charge protection. The battery in this RMCD device serves as a backup power supply when the node is not powered from the USB power supply. The battery, therefore, serves as a backup power supply that can maintain network operation of the entire monitoring system during mains power cuts. Especially when installing such monitoring systems in an environment where there are students, this solution seems to be very suitable. The battery in this RMCD device can operate for 24 to 36 h, depending on the data transmission period of the IQRF^®^ module. At 36 h of battery life, it is assumed that the measured data transmission period is every 5 min. Also, in battery mode, the indication of the measured CO_2_ concentration by the RGB LED is switched off. The measured CO_2_ concentration indication can be called up by pressing the user key “SHOW” on the RMCD. The power management block provides power adjustment for the TR-76 IQRF^®^ module and battery voltage switching for the DC/DC converter, CDM7160 carbon dioxide sensor and MCP9800 temperature sensor. The DC/DC boost block is a step-up module for powering the carbon dioxide concentration sensor and MCP9800 temperature sensor. The TR-76 block has an IQRF^®^ TR-76 transceiver. The Voltage Level Translator block is used to modify the voltage on the IIC bus and control the signals for the CDM7160 sensor. The HTU21 temperature and relative humidity sensor together with the MPL3115 atmospheric pressure sensor are connected to the IQRF^®^ TR-76 directly via the IIC bus.

The use of two temperature sensors is optional and depends on whether the user requires measurements of relative humidity and atmospheric pressure. If temperature measurement is sufficient, the less expensive MCP9800 may be used. RMCD devices have been installed in the authors’ department, which only allow monitoring of CO_2_ concentration and temperature using the MCP9800 temperature sensor. At the Grammar School and Secondary School of Electrical Engineering and Computer Science, Frenštát pod Radhoštěm, were used RMCD devices, which are able to measure CO_2_ concentration, temperature and relative humidity of ambient air and atmospheric pressure.

The CDM7160 carbon dioxide concentration sensor is a Non-Dispersive Infra-Red (NDIR) type. It works by reducing the intensity of a certain wavelength of infra-red light absorbed by CO_2_ molecules in the air. Specifically, it applies a differential spectral method. Air naturally (diffuses) flows into the sensor. IR light is used as a light source. IR spectrum selectivity is provided by a 4.3 μm wavelength filter in front of the detector. Drops in intensity are detected by an IR light detector. The FIGARO CDM7160 uses a second detector as a reference, which only detects a reference wavelength of 3.8 μm. Concentration is calculated according to intensity differences. The sensor is equipped with a non-woven particle filter. This filter reduces the effect of light scattering on particles and contamination of the optical system elements [27]. The advantage of using this sensor is that carbon dioxide concentration values are directly readable through the IIC bus or via the PWM output in the case of simpler circuits. Measurement accuracy is 50 ppm in the range 300–5000 ppm of CO_2_. Its disadvantage is increased consumption during measurement. Current consumption during measurement achieves up to 60mA. For this purpose, the sensor is switched off when there is no need to measure carbon dioxide concentration. A rough CO_2_ concentration value in certain intervals is also indicated by an RGB LED, as shown in Figure 1.

Permanently turning on the CDM7160 carbon dioxide sensor increases RMCD power consumption, which could then not last up to 36 h on a single battery charge. The RMCD is equipped with a battery to be able to monitor the environment even during a power outage. Switching off the CO_2_ concentration sensor is necessary to increase the battery operating time. The RMCD is designed to be able to measure and signal CO_2_ concentration without the need for communication in the IQRF^®^ network. This means that it acts autonomously as a mobile indicator of CO_2_ concentration at the installation site. In that case, the battery life of the RMCD is prolonged up to 100 h.

The main part of the RMCD device is an IQRF^®^ module manufactured by the Microrisc company, indicated in Figure 2 as TR-76. IQRF^®^ is a platform used for low speed, low power, reliable and easy-to-use wireless connectivity, for example, for telemetry, industrial control and building automation. It can be used with any electronic equipment and wherever wireless information transfer is needed in applications such as remote control, monitoring, alarms, displaying remotely acquired data or connecting several devices to a wireless network. IQRF^®^ is a complete system from a single brand that includes hardware, software, development support and services. IQRF^®^ transceivers can operate in worldwide ISM bands of 433MHz, 868MHz, and 916MHz. Data rate is limited to 19.2kb/s, but for automated data collection from sensors or for controlling actuators in home automation, this is sufficient. The IQRF^®^ consists of an 8-bit microcontroller and RF chip with additional hardware such as EEPROM memory, LED, and temperature indicator. Several types of IQRF^®^ modules are available. The TR-76 module has a minimum of additional components, which reduces power consumption. Its additional advantage is extra input/output ports. Communication is packet-oriented and allows a maximum payload of 64B per packet. The maximum number of nodes in one IQRF^®^ network is 240 devices, including the coordinator [7,28]. In the article [29] which deals about smart home monitoring system, they use XBee technology for individual sensors. At the end of this article, the authors argue that by replacing this communication technology with IQRF^®^ technology, they have managed to reduce the power consumption of each wireless sensor node by 20% at the same coverage. As a further advantage, they highlight the wider scalability of the system based on this technology. One of the case studies using IQRF^®^ technology is monitoring of cattle in outdoor environment—see [30]. The article [31] briefly describes the genesis of IQRF^®^ technology and shows a simulation of signal propagation in IQMESH network in various environments. Finally, it provides a comparison with other wireless communication technologies such as LoRa WAN, ZigBee, BLE, Wi-Fi MESH HaLow and LoRaWAN. The article [32] gives a comparison of IQRF^®^ technology with LoRa and Sigfox technologies in terms of data throughput and collisions in transmitting messages. Based on the findings and the authors’ own experience, IQRF^®^ technology is ideal for the implementation of the Internet of Things in the field of monitoring of environmental quantities.

MESH topology involves connecting each node to all others (full mesh), thereby providing high reliability. If any of the routes become unavailable, an alternative path to deliver messages (usually multiple paths) can therefore be formed.

In a wireless IQRF^®^ network using mesh topology, each node has a defined time transmission slot, which ensures transmission does not interfere with other nodes. Nodes continuously receive and forward messages during their transmission slots to all nearby devices. If multiple nodes are somehow damaged, messages have a high probability of arriving at the destination due to the presence of alternative paths. Routing in IQRF^®^ networks is based on directional flooding of the network and TDMA (Time division multiple access) [33]. An example of how the communication among IQRF^®^ nodes works is shown in animation [34].

IQMESH is a protocol for wireless IQRF^®^ networks developed by the Microrisc company. It is based on mesh topology, which ensures better coverage and a broader range for IQRF^®^ transceivers deployed at a location. Neighbors are transceivers that are mutually within the transmission range.

If, for example, 15 transceivers are deployed in an area using mesh topology, each of them transmits during its own time interval. In this case, this will be maximum of 15 intervals. Transceivers transmit in a synchronized manner and therefore they do not interfere with each other during transmission.

**Figure 3:**
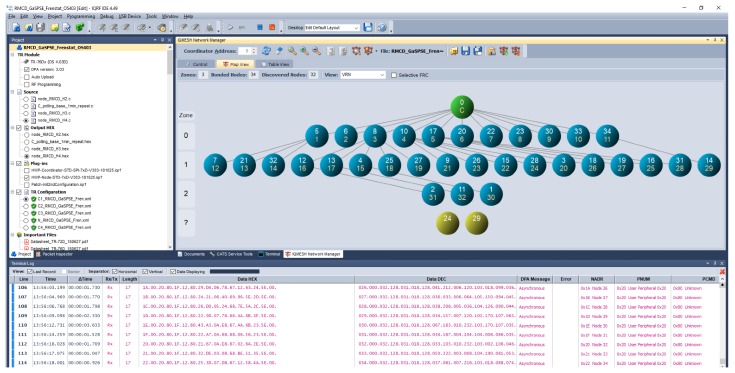
The structure of the discovered IQMESH network in the main building of the Grammar School and Secondary School of Electrical Engineering and Computer Science, Frenštát pod Radhoštěm.

Current situation of installing 34 RMCD sensors in the main building of the Grammar School and Secondary School of Electrical Engineering and Computer Science, Frenštát pod Radhoštěm shows Figure 3.

Figure 3 shows that the maximum number of jumps (routes) from the network coordinator to the furthest IQMESH node of the network is three. The outermost network nodes are located in zone 2, they are RMCD nodes No. 1, 2 and 11. In Figure 3 you can also see the “undiscovered” network nodes No. 24 and 29, for which the IQMESH network coordinator sets the maximum number jumps to the addressed sensor to the maximum number of nodes in the network.

The total timeout time can be calculated according to the formula:

$$\begin{array}{c} Timeout=(jumps\;C\to N+jumps\;N\to C+2\;additional\;jumps)\cdot TimeSlot\\ +\;extra\;time\;for\;data\;preparing\;in\;RMCD\;node \end{array}$$

The length of the *TimeSlot* depends on the size of the data message and the type of IQMESH network (standard mode or low-power mode). This *TimeSlot* is controlled by the operating system of the IQRF^®^ module itself and in this case it is 90 ms. It follows that the “undiscovered” nodes No. 24 and 29 will have a timeout for their response:

Timeout=(34·+34+2)·0.09+2=8.3s

This timeout is only a recommendation for the superior system not to send a new data request before a response to the previous request arrives. The way of addressing the undiscovered node is done by the “flooding” method [24]. The main control transceiver—the IQMESH network coordinator—dispatches data when required. Nearby transceivers receive these data and progressively forward them to the neighborhood during their time intervals. Other transceivers then receive this data and again progressively forward it during their time intervals, spreading the data throughout the network in this manner [7,35]. Due to the robustness of the IQMESH network, however, a message from an undiscovered network node is delivered in approximately 3 s, as shown in the Terminal Log window in Figure 3 below.

### 2.2. Measurement Network

The proposed measurement network consists of wireless RMCD measuring nodes and a central unit. The quantities measured are CO_2_ concentration, temperature and relative humidity in the surrounding air and atmospheric pressure. The sensors have a digital output with communication via an IIC bus. Data measured by these sensors are sent through IQRF^®^ transceivers, or nodes [7].

**Figure 4:**
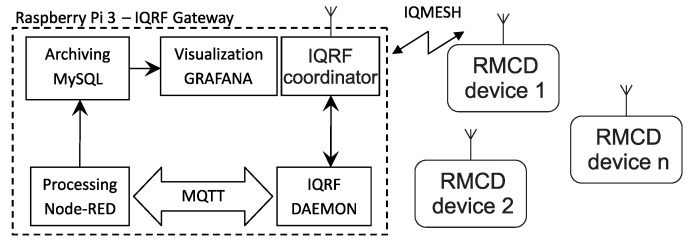
Scheme for processing, archiving, and visualizing measured data.

The measured data can be processed and visualized on a Raspberry Pi3 microcomputer [4]. The IQMESH network coordinator can be connected to up to 239 wireless measurement nodes consisting not only of RMCD measuring modules but also other types of sensors capable of connecting to IQMESH. The basic structure of a Raspberry Pi system with the required applications installed is shown in Figure 4.

Measured data are accepted from the IQMESH network coordinator by the IQRF^®^ Daemon application and subsequently transferred via the MQTT service to an application in the Node-RED environment. Data processed in this way are already recorded in a ready-to-read format and transferred to the local MySQL database [36]. The stored data are visualized using the Grafana platform. Data from individual RMCD devices is sent via DPA packets. Direct Peripheral Access (DPA) protocol is a simple byte-oriented protocol used to control IQMESH network services (Coordinator and Nodes) [26,37]. For easier work with the database and visualization, this DPA packet is converted into a so-called ready-to-read format. This means that the ready-to-read format is the form of a message that is directly readable by a human. This shape is easy to store in a database and then visualized in the Grafana platform.

In the cases presented in these studies [2,3], all processing and visualized components ran simultaneously on a single board computer. However, using separate computers (servers) for individual services is also possible.

IQRF^®^ Daemon establishes communication between the IQMESH network coordinator and other systems via UDP, MQTT or WebSocket. The application can be connected to the IQRF^®^ IDE development environment (see Figure 3) through a UDP channel. The IQRF^®^ Daemon application has a superstructure with a web interface for configuring and controlling the IQMESH network directly from a web browser. The application is designed to be modular and open source.

Node-RED is a graphical programming environment based on JavaScript. Measured data are received via the MQTT protocol, then processed and stored in a database. The advantage of this solution is high speed implementation and easy modification or simple extension.

The measured data are received in raw form via MQTT as JSON text. The collected data are then processed. Processing includes converting CO_2_ concentrations, temperatures, relative humidities and atmospheric pressures of all polled sensors. For simplicity, a custom *RMCD* data processing block has been created. Data collected in this manner are then used to compile the SQL query through which the MySQL database is stored. All data processed in this manner have unique identifiers in the database and contain timestamps and NODE identifiers.

Grafana is a visualization tool for web interfaces. Visualizations for measured data can be created using the Grafana platform. Users can browse information in historical graphs, or they can inspect current or recently measured values.

As mentioned above, data from RMCD sensors measuring CO_2_ could be analyzed on a Raspberry Pi3 microcomputer. However, where a reliable wireless sensor network is required, using a dedicated IQRF^®^/Ethernet gateway was more suitable. The device transmitted the measured CO_2_ concentrations to a cloud as raw data. These data were then processed on the server side located at VŠB-TUO. The Grafana visualization software also ran on this server and was used to monitor the levels of CO_2_ concentration, temperature, relative humidity, and atmospheric pressure from any place with internet access.

The measurement network consists of four main components:

1. RMCD device—transmits measured CO_2_ concentration, temperature and relative humidity of ambient air, and atmospheric pressure data upon request.
2. IQRF^®^/Ethernet gateway contains a network coordinator, microcontroller and Ethernet interface for internet connectivity. This device works in two modes either as a datalogger or a gateway. In datalogger mode, all data received from an IQRF^®^ network is sent to the cloud, or vice versa, data are transmitted from the cloud to the IQRF^®^ network. Gateway mode allows messages to be transmitted from the IQRF^®^ network to the IQRF^®^ IDE development environment. This mode enables the remote setting of IQRF^®^ network parameters, such as adding or removing network nodes. The gateway also works with the UDP channel, providing communication with the IQRF^®^ IDE development environment as well as an MQTT broker with the Node-RED tool.
3. IQRF^®^ Cloud—a commercial service that collects IQRF^®^ data from IQRF^®^/GSM or IQRF^®^/Ethernet gateway. This cloud enables bi-directional data transfer, i.e., from the gateway to the cloud and the cloud to the gateway. The gateway must be set in datalogger mode.
4. Visualization—measured data are visualized using Grafana software. This SW is installed on the VŠB-TUO server. The basis of each visualization tool is a data source. In our case studies, we have used the MySQL database, which was also installed on the university server enviro.vsb.cz [38] (login: sensors, password: sensors). For each IQRF^®^/Ethernet gateway, a table is created in the database where processed data from the IQRF^®^ cloud are stored. Grafana is also accessible from outside the university network, therefore dashboards can be viewed from anywhere as long as access to the Internet is available.

An integral part of the IQRF^®^/Ethernet gateway is the IQRF^®^ network coordinator. The network coordinator contains a custom DPA handler, which is a program that automatically sends commands to read all measured data from individual RMCD devices. The reading period of the sensors can be set in the coordinator’s EEPROM. The time interval can range from one minute up to 255 min. In this study, the time interval was set to 5 min. Another parameter of this program is the maximum number of queried nodes and the number of query repetitions if the coordinator did not receive a response from the requested network node.

**Figure 5:**
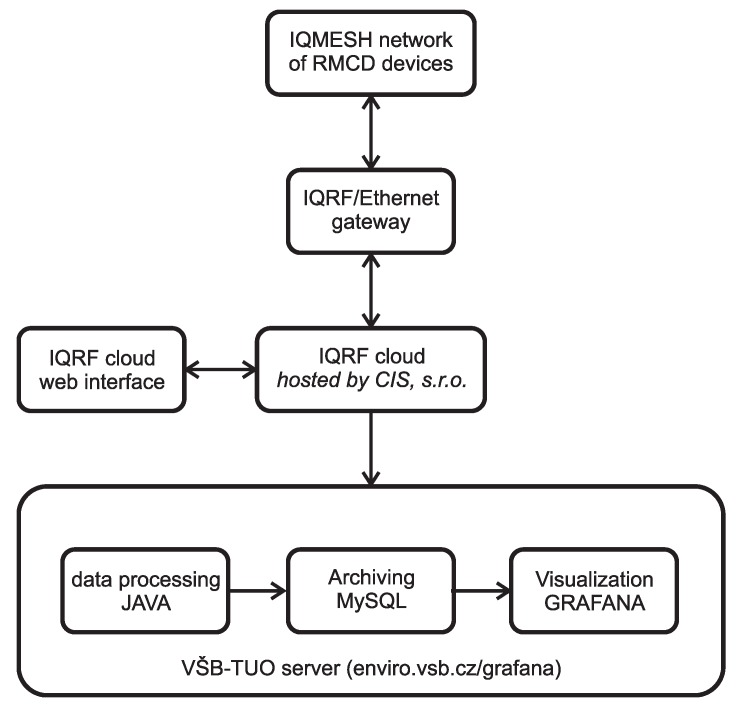
Scheme for processing, archiving, and visualizing measured data for case studies.

Data processing of measured quantities takes place on the side of the university server, see Figure 5.

Measured data in raw format are sent to the IQRF^®^ cloud via the IQRF^®^/Ethernet gateway. On the university server enviro.vsb.cz runs a software utility created in the programming language JAVA. This utility queries the IQRF^®^ cloud for measured data cyclically using HTTP request with a period of 1 min. The raw data are then converted into individual values of measured quantities using this utility and these measured quantities are then stored in the appropriate table in the MySQL database. The database then serves as a data source for visualization using the Grafana SW system. If it is not necessary to use a commercial IQRF^®^ cloud, the solution shown in Figure 4 can be used, where the conversion of raw data from individual sensors is done in Node-RED.

## 3. Analysis of Carbon Dioxide Measurements

The next part of the text will analyze the CO_2_ concentration data in selected rooms at VŠB—Technical University of Ostrava and at Grammar School and Secondary School of Electrical Engineering and Computer Science, Frenštát pod Radhoštěm. The graphs of other monitored quantities, i.e., temperature and relative humidity of the ambient air in individual monitored rooms, are a part of Appendix A of this article. These data show the effect of ventilation on the CO_2_ concentration. With the window open, the room temperature decreases, and the relative humidity also decreases. Thus, there is a correlation between the measured CO_2_ concentration, temperature, and relative humidity of the ambient air.

### 3.1. Case Study—Department of Cybernetics and Biomedical Engineering, VŠB-Technical University of Ostrava

The pilot operation was analyzed to learn about the amount of time students and staff spent in an environment that could influence their study or work performance. From the data analysis, modifications to ventilation or recuperation could then be recommended. For this purpose, a network of eight RMCD sensors with wireless transmission in an IQRF^®^ network was created.

**Figure 6:**
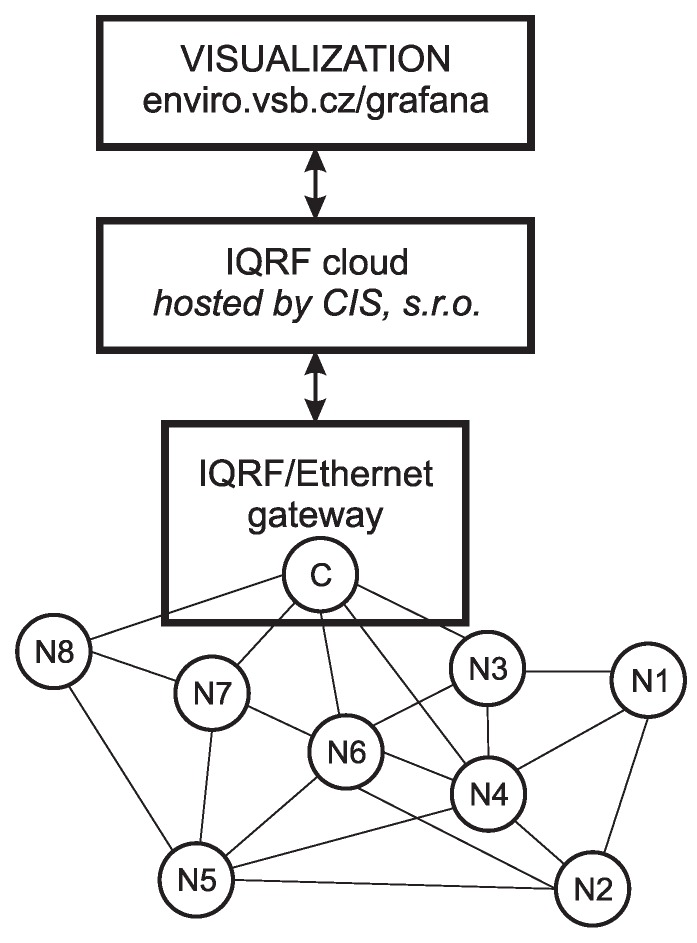
Block scheme for monitoring the environmental quantities at the Department of Cybernetics and Biomedical Engineering at VŠB-TUO.

Figure 6 shows a block diagram of this network.

These sensors were installed in departmental laboratories and offices in the same workplace.

**Figure 7:**
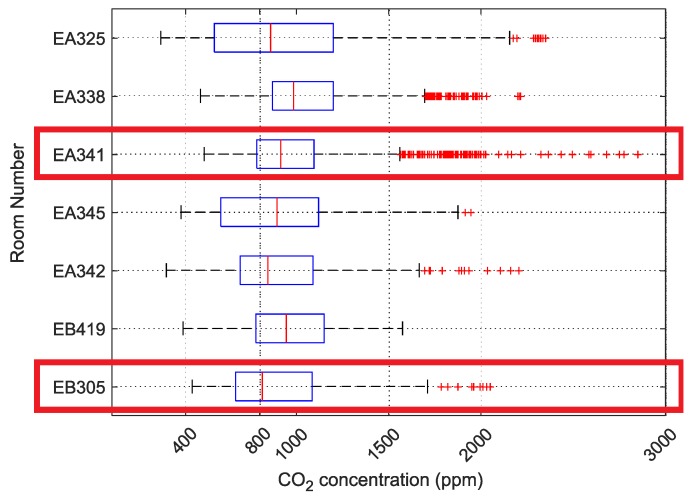
Horizontal boxplot for the February 2019 at the Department of Cybernetics and Biomedical Engineering, VŠB-TUO; The red boxes mean the rooms that are further analyzed in this case study.

The period 7:00 AM to 5:00 PM was selected (which corresponds to 120 values/time range, with a 5-min measurement period) for this study. The results were summarized without taking into account the individual laboratory schedules and working hours of individual employees in their offices. Data outliers, i.e., values greater than 5500ppm, were removed from the dataset. A boxplot consisting of these data was created for the month of February 2019 (Figure 7).

**Figure 8:**
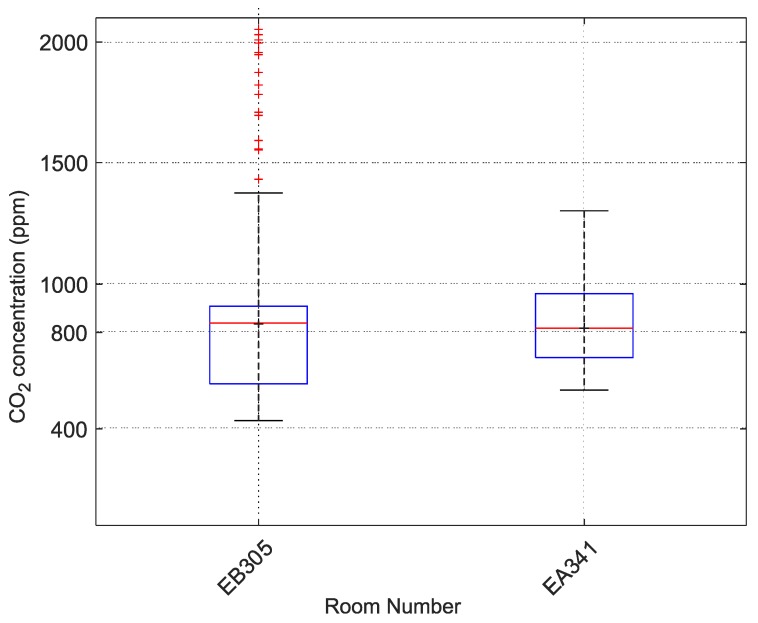
Boxplot for room numbers EA341 and EB305 on 18 February 2019, VŠB-TUO.

Datasets were selected according to the horizontal boxplot in Figure 7 and the measured values were graphed according to the schedule or work time. Room number EA341, which had two employees, and laboratory EB305, where approximately 20 students were taught, were selected, with 18 February as the date. The boxplot for this day at these two locations is given in Figure 8. This section of the case study examines these two rooms in more detail.

**Figure 9:**
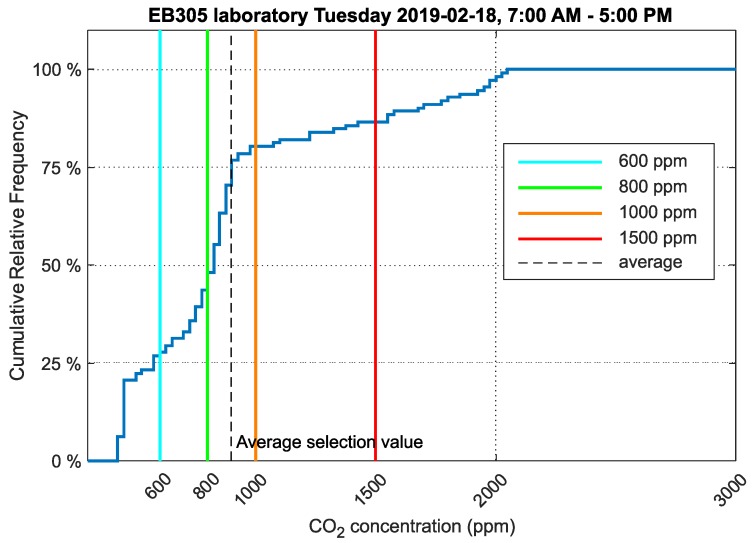
Cumulative relative frequency graph of the data selection showing CO_2_ concentration in laboratory EB305, VŠB-TUO.

These data were analyzed using cumulative frequency graphs (Figure 9). These graphs show in synoptic form when the selected time period had CO_2_ concentrations less than the selected limit values. The same procedure is also possible for other analyzed quantities [2].

The data were analyzed according to the methodology defined for measurement and evaluation in this study. The vertical lines in the graph show CO_2_ concentration levels. The light blue line shows 600ppm CO_2_ concentration, the green line 800ppm, orange 1000ppm and red 1500ppm. These vertical lines divide the cumulative frequency diagram into five areas where the values below the light blue line indicate how much of the monitored time was spent in a room where the CO_2_ concentration was less than 600 ppm.

The values measured in laboratory EB305 on 18 February 2019 during teaching hours (7:00 AM–5:00 PM) were analyzed. Approximately 20 persons were situated in this laboratory during teaching hours.

The cumulative frequency graph for CO_2_ concentration (Figure 9) shows that the values did not exceed the limit of 1500ppm in 86% of the time people were present in the room.

**Figure 10:**
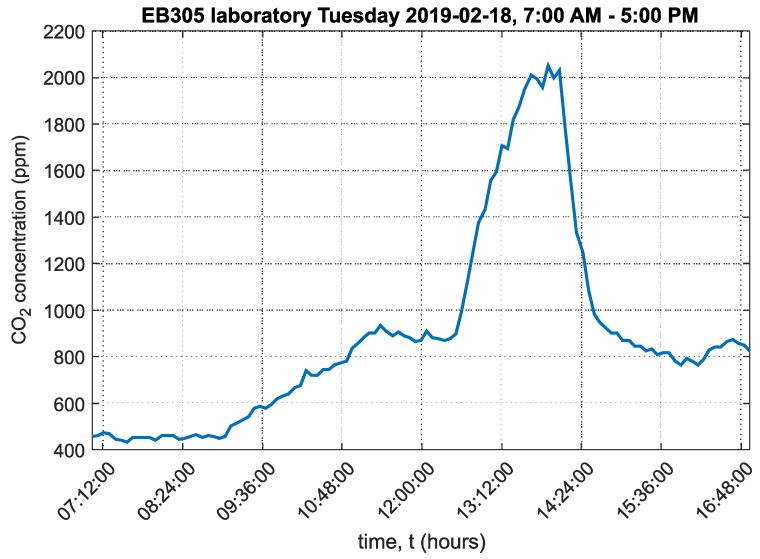
Graph of carbon dioxide concentration in laboratory EB305, VŠB-TUO.

A chart of the carbon dioxide concentration over the course of the day is shown in Figure 10.

**Figure A1:**
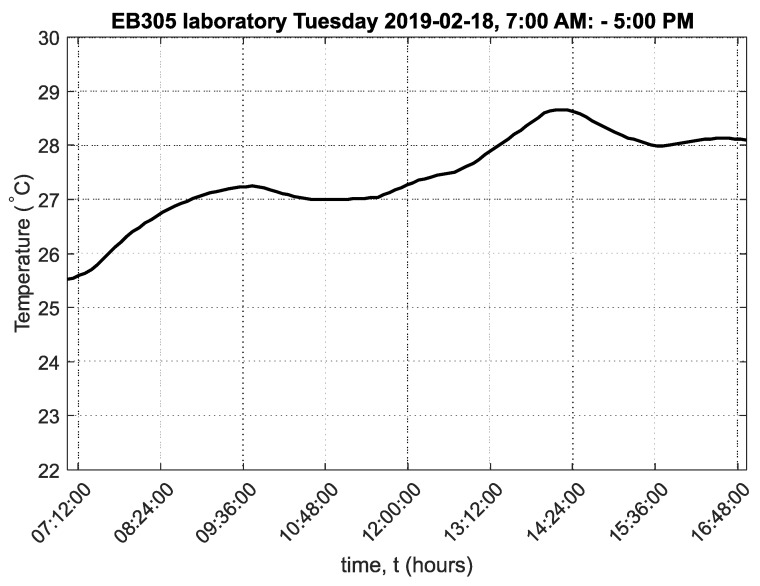
Graph of ambient temperature in laboratory EB305, VŠB-TUO.

The graph shows that carbon dioxide concentration in the room peaked at around 2200ppm as a result of no ventilation to the laboratory. In the first section of the chart, one window from three was open. The increase in CO_2_ concentration means that ventilation was insufficient. In the second section of the chart, after lunch time, the windows were closed, and CO_2_ concentration levels rose rapidly. After the lessons, all three windows were opened for 20 min. The ambient temperature chart in EB305 laboratory is shown in Appendix A in Figure A1.

**Figure 11:**
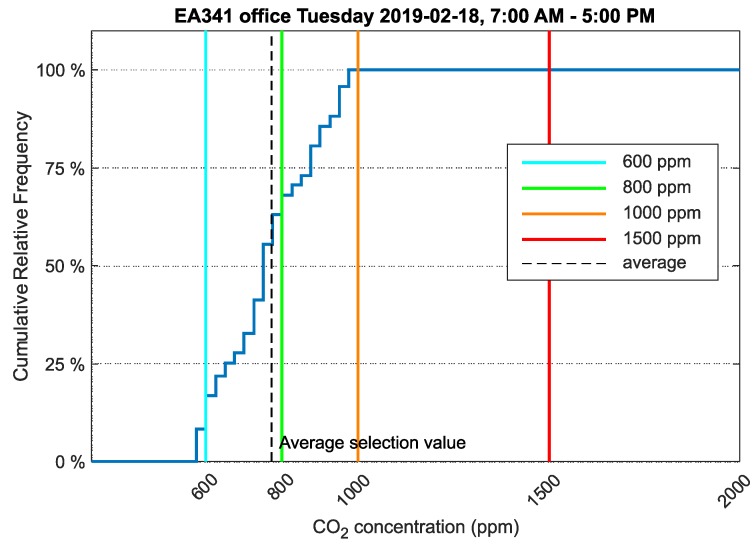
Cumulative relative frequency graph of the data selection showing CO_2_ concentration in office EA341, VŠB-TUO.

The environment in laboratory EB305 was compared to a standard office room with two employees. Office EA341 had the same ventilation system as the laboratory. A relative cumulative frequency graph of CO_2_ concentration for office EA341 is presented in Figure 11.

The values measured in office EA341 for the same date and time interval as laboratory EB305 were analyzed. Two employees were present throughout the workday.

The relative cumulative frequency graph for CO_2_ concentration (Figure 11) shows that the values did not exceed the limit of 800ppm in 68% of the time the employees were present. This, therefore, suggests a more comfortable environment in direct contrast to the EB305 laboratory.

**Figure 12:**
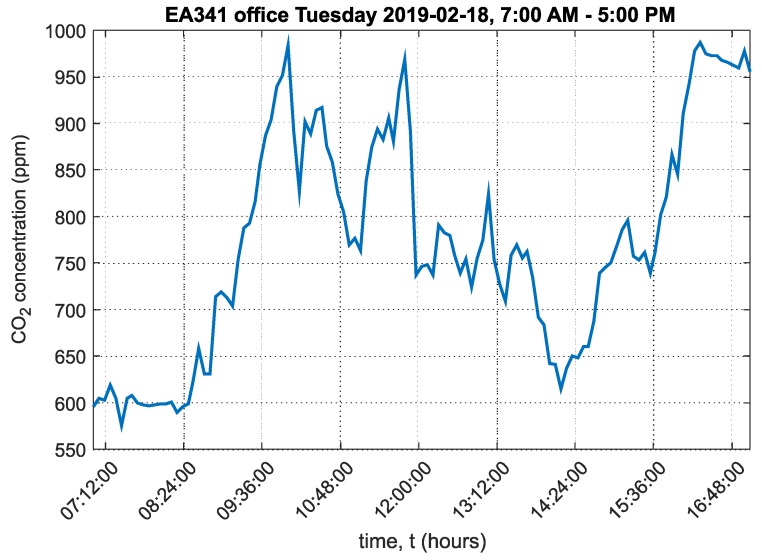
Graph of carbon dioxide concentration in office EA341, VŠB-TUO.

A graph of the carbon dioxide concentration over the course of the day in office EA341 is shown in Figure 12.

**Figure A2:**
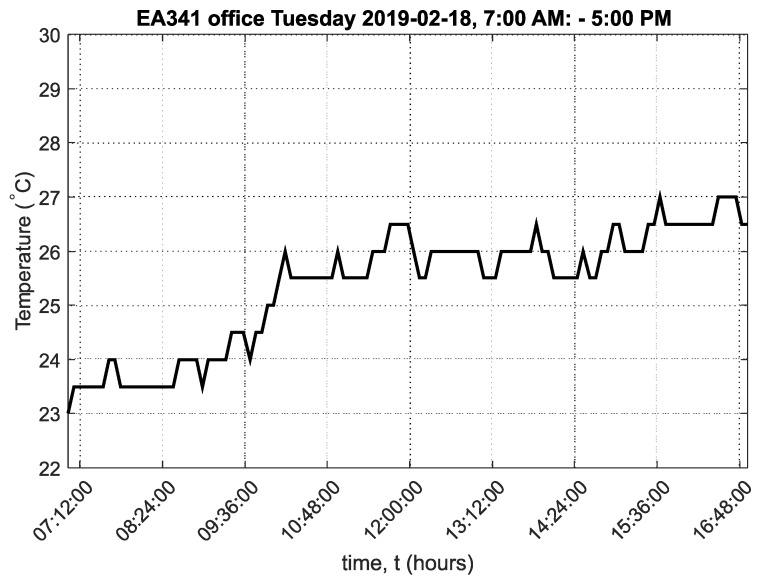
Graph of ambient temperature in office EA341, VŠB-TUO.

The graph shows that the maximum CO_2_ concentration did not exceed 1000ppm. This was due to the window being open throughout the day. At the end of the workday, it is clear that CO_2_ concentration was slow to decrease. The ambient temperature chart in EA341 office is shown in Appendix A in Figure A2.

### 3.2. Case Study–Grammar School and Secondary School of Electrical Engineering and Computer Science, Frenštát pod Radhoštěm

The second case study examined the installation of a larger number of measuring sensors in classrooms and high school laboratories. This installation followed the pilot operation at the Department of Cybernetics and Biomedical Engineering of VŠB-TUO.

**Figure 13:**
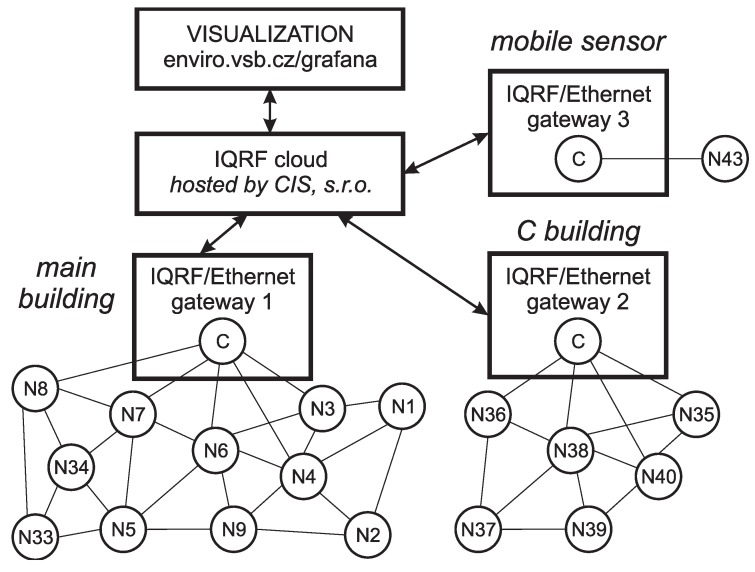
Block scheme for monitoring the environmental quantities at the Grammar School and Secondary School of Electrical Engineering and Computer Science, Frenštát pod Radhoštěm.

For this purpose, a network of 40 RMCD sensors with wireless transmission in an IQRF^®^ network was created. The block diagram is shown in Figure 13.

The measurement system was deployed in two buildings. Thirty-four RMCD measurement nodes (nodes N1 to N34) were placed in the main building. These nodes were queried periodically by the IQRF^®^/Ethernet gateway coordinator No. 1. The building indicated by the letter “C” had six sensors (nodes N35 to N40). These nodes were periodically queried by the coordinator in the IQRF^®^/Ethernet gateway No. 2. Two of the RMCD measurement nodes were used for high school student teaching purposes (designated N41 and N42). These nodes were not permanently enabled. Measurement node N43 was wirelessly connected to the IQRF^®^/Ethernet gateway No. 3, and these two components served as a mobile sensor for deployment anywhere in the high school premises. Measurement nodes N41 and N42 were connected to training kits with a similar configuration to that shown in Figure 4. The only difference from the scheme in Figure 4 is that instead of a Raspberry Pi3 microcomputer, an UpBoard computer was used, which has better operating parameters and a more robust design.

**Figure 14:**
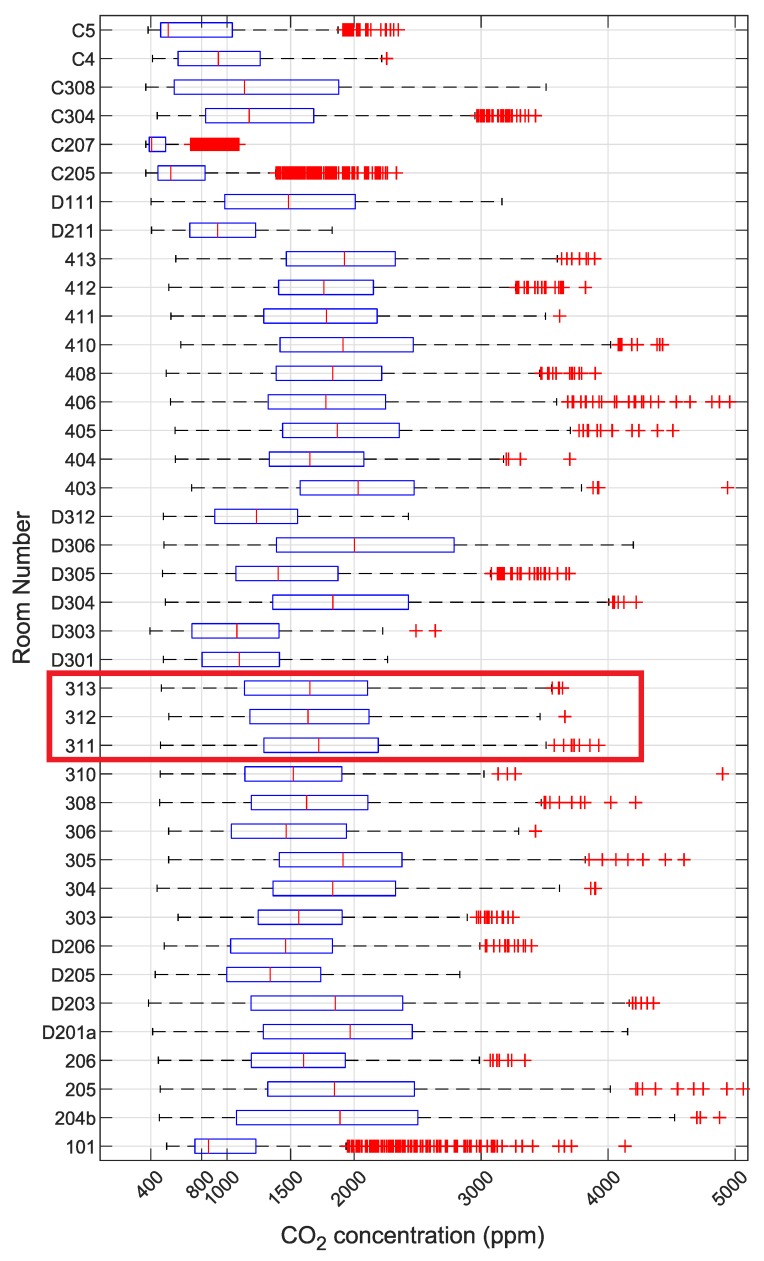
Horizontal boxplot for the January 2019 at the Grammar School and Secondary School of Electrical Engineering and Computer Science, Frenštát pod Radhoštěm; The red box means the classrooms that are further analyzed in this case study.

The weekday period 7:00 AM–3: 00 PM was selected (which corresponds to 84 values/time range, with a 5-min measurement interval) for this study. The results were summarized without taking into account the individual room schedules. Data outliers, i.e., values greater than 5500ppm, were removed from the dataset. A boxplot consisting of these data were created for the month of January 2019 (Figure 14).

Datasets were selected according to the horizontal boxplot in Figure 14 and the measured values were graphed according to the schedule. Classrooms 311, 312 and 313 for 15 January 2019 were selected for further analysis. Table 2 summarizes the student teaching schedule for this day.

**Figure 15:**
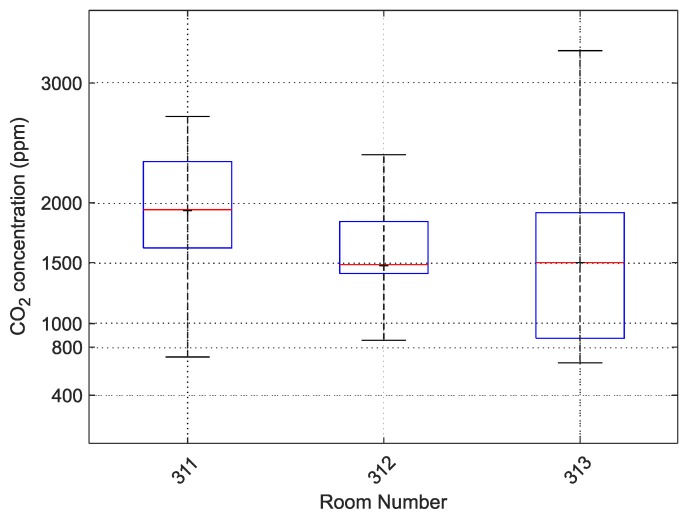
Boxplot for classrooms 311, 312 and 313 on 15 January 2019, Grammar School and Secondary School of Electrical Engineering and Computer Science, Frenštát pod Radhoštěm.

The boxplot for these three rooms for the given calendar day is shown in Figure 15. This section of the case study examines these three rooms in more detail.

**Figure 16:**
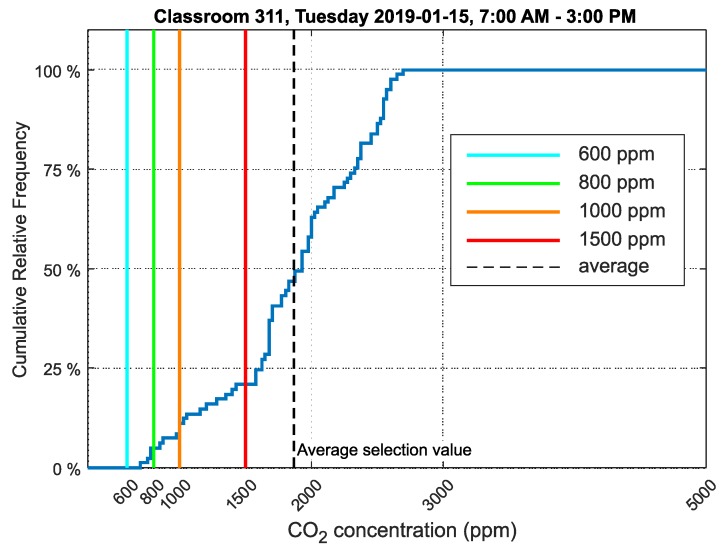
Cumulative relative frequency graph of the data selection showing CO_2_ concentration in classroom number 311, Frenštát pod Radhoštěm.

A cumulative relative frequency graph for classroom 311 is shown in Figure 16.

The measured data were analyzed according to the methodology defined for measurement and evaluation in this study. The limit values are marked as a color lines. The explanation is mentioned under Figure 9 in Section 3.1.

The values measured in classroom 311 on 15 January 2019 during the teaching schedule (7:00 AM–3:00 PM) were selected for analysis. On this day, second, fourth and sixth graders of the high school were in the classroom (Table 2).

The relative cumulative frequency graph for CO_2_ concentration (Figure 16) shows that the values did not exceed the limit of 1500ppm in 21% of the time students were present in the room.

**Figure 17:**
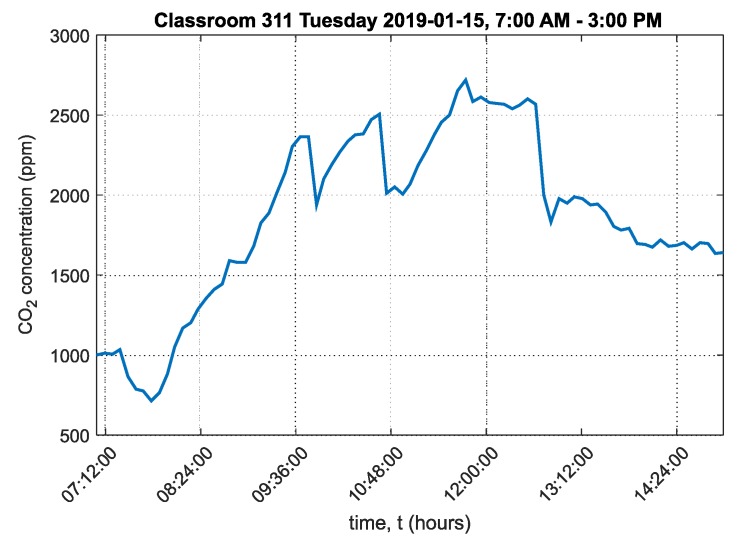
Graph of carbon dioxide concentration in classroom 311, Frenštát pod Radhoštěm.

A graph of the carbon dioxide concentration over the course of the day is shown in Figure 17.

**Figure A3:**
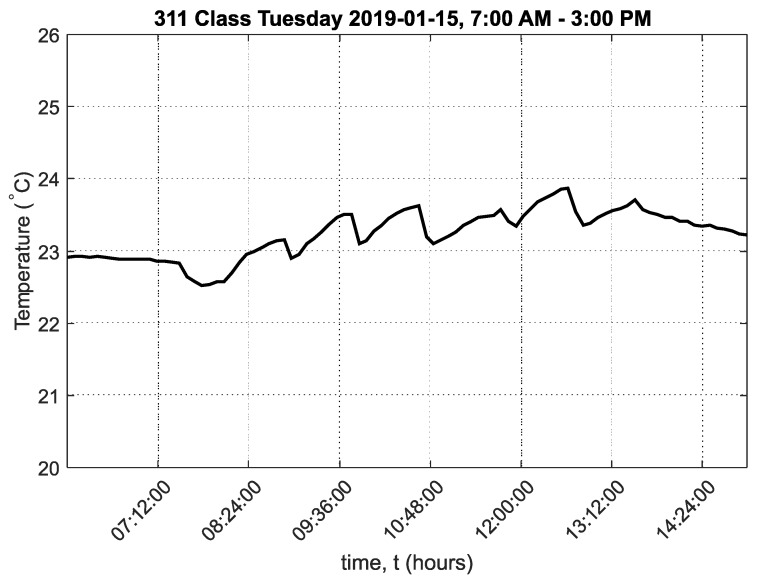
Graph of ambient temperature in classroom 311, Frenštát pod Radhoštěm.

**Figure A4:**
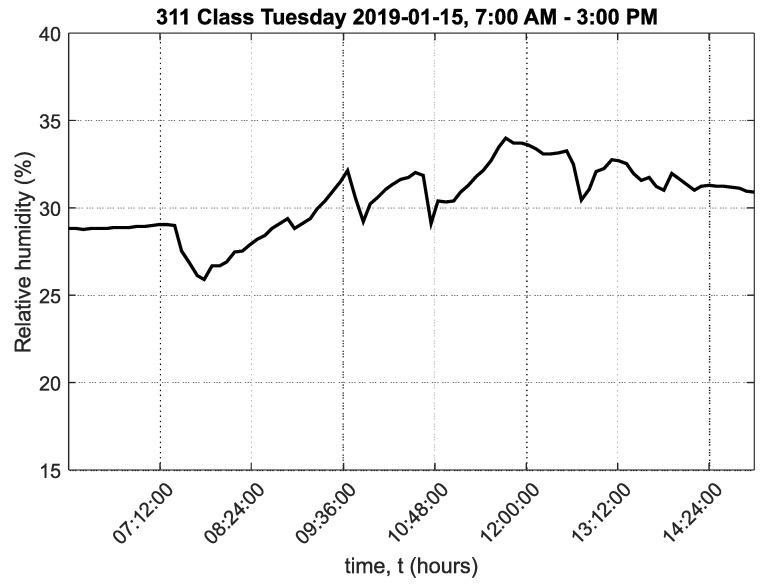
Graph of relative humidity of ambient air in classroom 311, Frenštát pod Radhoštěm.

The graph shows that the carbon dioxide concentration in the room peaked at around 2700ppm. The graph also shows that the room was only ventilated during breaks, which was insufficient since CO_2_ concentration did not drop below 1500ppm after ventilation. The ambient temperature chart in classroom 311 is shown in Appendix A in Figure A3. The ambient relative humidity chart in classroom 311 is shown in Appendix A in Figure A4.

**Figure 18:**
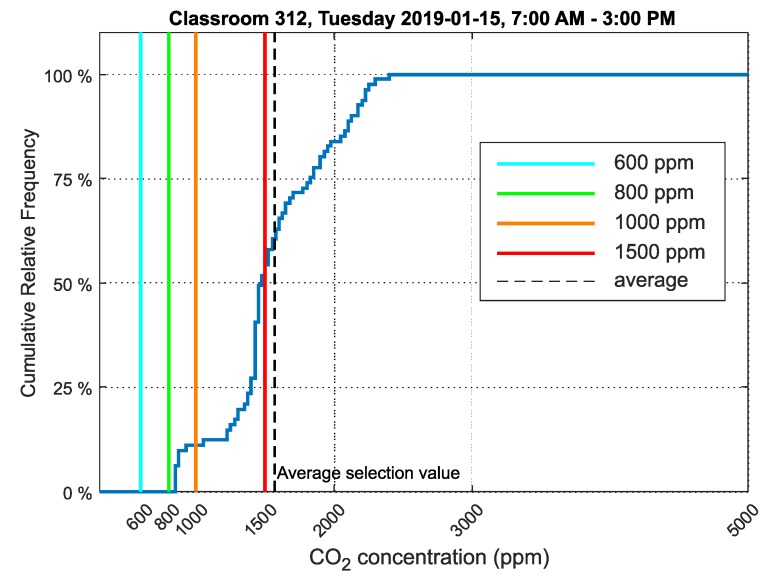
Cumulative relative frequency graph of the data selection showing CO_2_ concentration in classroom 312, Frenštát pod Radhoštěm.

In classroom 312, seventh and third grade students were taught in the morning, and fifth and second graders were taught in the afternoon. A relative cumulative frequency graph of CO_2_ concentration for classroom 312 is presented in Figure 18.

The values measured in classroom 312 on the same date and time interval as classroom 311 were selected for analysis.

The relative cumulative frequency graph for CO_2_ concentration (Figure 18) shows that the values did not exceed the limit of 1500ppm in 52% of the time students were present in the room, which suggests a better environment than classroom 311.

**Figure 19:**
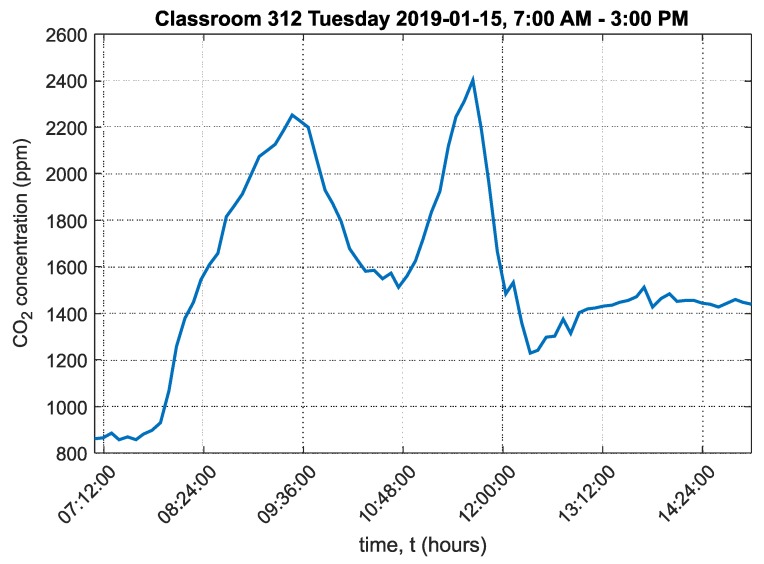
Graph of carbon dioxide concentration in classroom 312, Frenštát pod Radhoštěm.

A graph of the carbon dioxide concentration over the course of the day in classroom 312 is shown in Figure 19.

**Figure A5:**
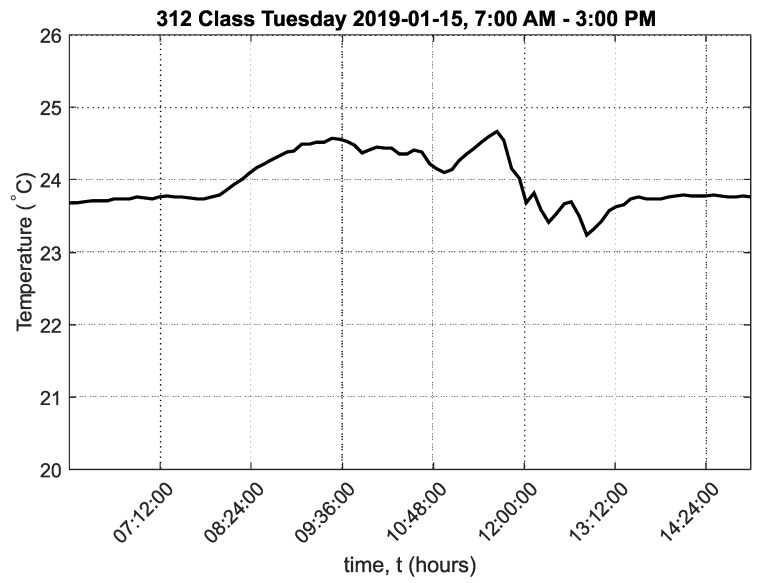
Graph of ambient temperature in classroom 312, Frenštát pod Radhoštěm.

**Figure A6:**
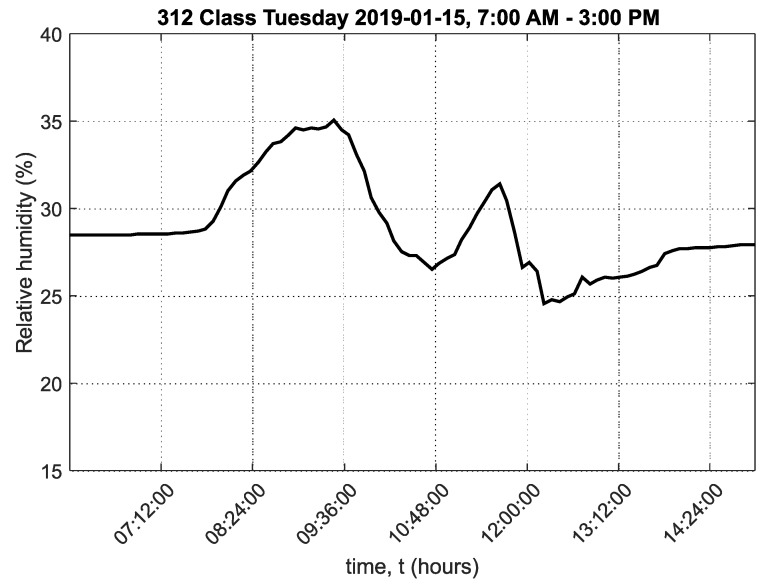
Graph of relative humidity of ambient air in classroom 312, Frenštát pod Radhoštěm.

This graph shows that the maximum CO_2_ concentration significantly exceeded 1500ppm twice over the course of the day. The maximum CO_2_ concentration reached 2400ppm. The ambient temperature chart in classroom 312 is shown in Appendix A in Figure A5. The ambient relative humidity chart in classroom 312 is shown in Appendix A in Figure A6.

**Figure 20:**
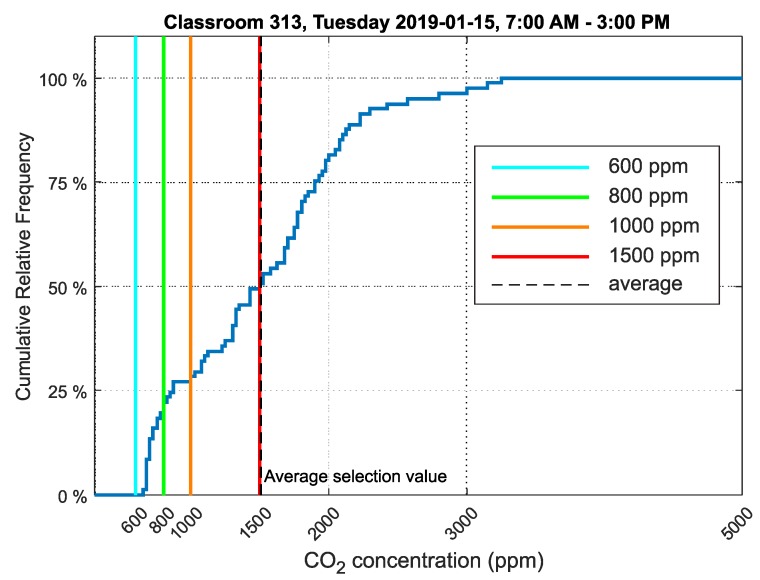
Cumulative frequency graph of the data selection showing CO_2_ concentration in classroom 313, Frenštát pod Radhoštěm.

The final classroom studied was classroom 313. In this classroom, second year students were taught in the morning. A free lesson without teaching followed, and in the afternoon, lessons for fifth and eighth graders were held until 2:20 PM. A relative cumulative frequency graph for CO_2_ concentration for classroom 313 is presented in Figure 20.

The values measured in classroom 313 on the same date and time interval as classrooms 311 and 312 were selected for analysis.

The relative cumulative frequency graph of CO_2_ concentration (Figure 18) shows that the values did not exceed the limit of 1500ppm in 50% of the time students were present in the room, indicating a worse environment to classroom 311. Compared to the cumulative relative frequency chart for classroom 312 (Figure 18), the environment in classroom 313 was similar.

**Figure 21:**
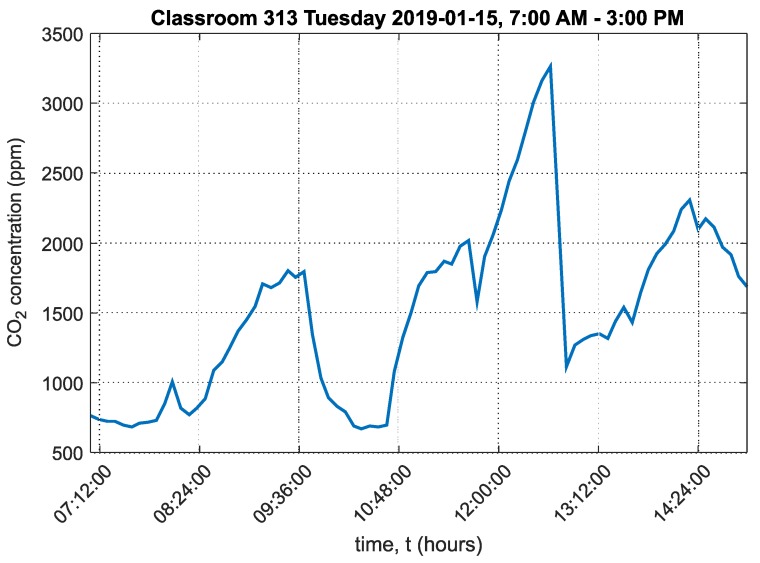
Graph of carbon dioxide concentration in classroom 313, Frenštát pod Radhoštěm.

A graph of the carbon dioxide concentration over the course of the day in classroom 313 is shown in Figure 21.

**Figure A7:**
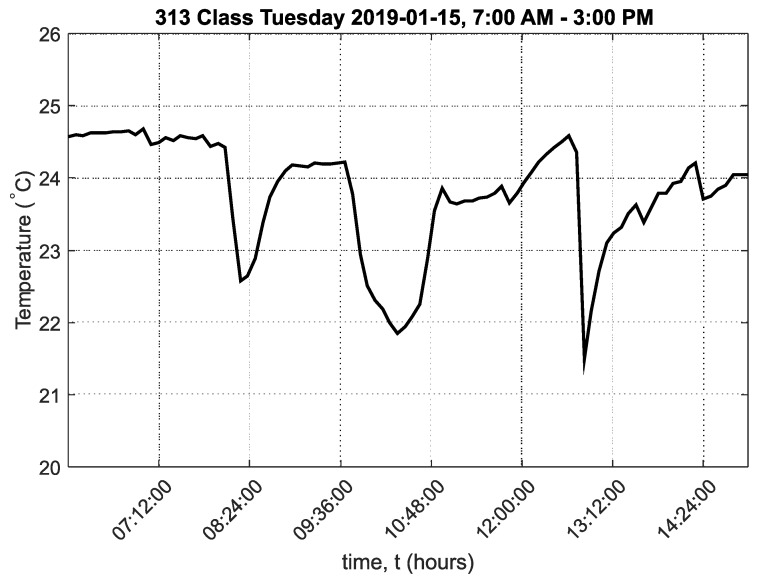
Graph of ambient temperature in classroom 313, Frenštát pod Radhoštěm.

**Figure A8:**
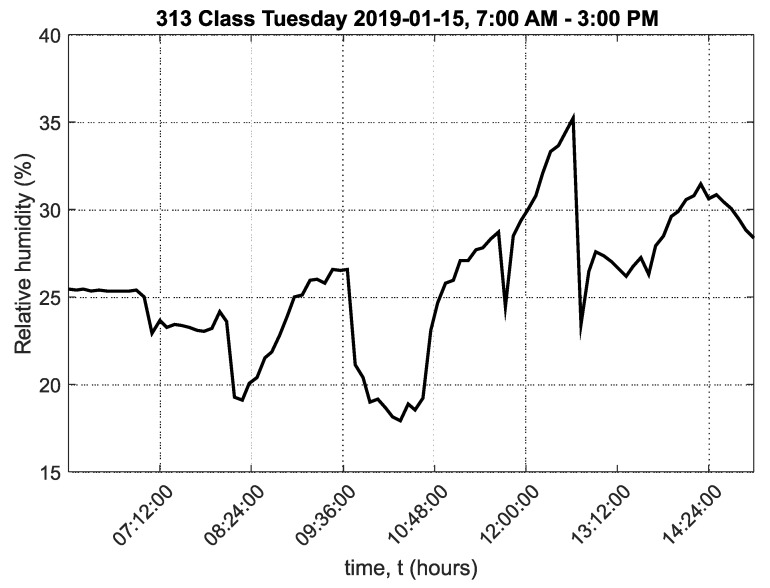
Graph of relative humidity of ambient air in classroom 313, Frenštát pod Radhoštěm.

The graph shows that the maximum CO_2_ concentration significantly exceeded 1500ppm and reached 3263ppm. In the morning, CO_2_ concentration exceeded 1500ppm for only about one hour. At this time, second year students were present in the classroom. In the afternoon, when the second, fourth and eighth graders were taught, CO_2_ exceeded 1500ppm significantly and reached the abovementioned maximum value. This occurred twice over the course of the day. The ambient temperature chart in classroom 313 is shown in Appendix A in Figure A7. The ambient relative humidity chart in classroom 313 is shown in Appendix A in Figure A8.

## 4. Results

The analysis results of both case studies are summarized in the following two subsections.

### 4.1. Department of Cybernetics and Biomedical Engineering, VŠB-Technical University of Ostrava

One office and one laboratory were selected, and the environments were compared. The analyzed data were from 18 February 2019, i.e., winter when outdoor temperatures did not exceed 0°C and therefore ventilation time is minimized. The mean CO_2_ concentration value in laboratory EB305 was 897ppm. The mean CO_2_ concentration value in office EA341 was 773ppm. This is only a difference of 124ppm, but when the data were analyzed as relative cumulative frequency of CO_2_ concentration, the results were more significant. In laboratory EB305, students remain exposed to 1500ppm of CO_2_ concentration.

In laboratory EB305, students spent 14% of teaching time in an environment with a CO_2_ concentration of 1500ppm or more. Worse results were obtained from the analysis of time spent by students in an environment above 800ppm of CO_2_. In this environment, students were exposed for more than 52% of their teaching time. In office EA341, the two employees spent 32% of their work time in an environment with CO_2_ concentrations above 800ppm. A comparison for 1500ppm of CO_2_ concentration could not be made because CO_2_ concentration in EA341 did not exceed 1000ppm of CO_2_ over the analyzed period.

Table 3 summarizes the statistical information of the data collected for the period 7:00 AM–5:00 PM.

### 4.2. Grammar School and Secondary School of Electrical Engineering and Computer Science, Frenštát pod Radhoštěm

Data sets for three classrooms where students from second to eighth grade are taught were statistically analyzed. The students distributed their time spent in individual rooms. The data were collected on 15 January 2019, when external temperatures were below freezing. In classrooms 312 and 313, the average CO_2_ concentration values were approximately the same. Class 312 recorded 1567ppm and classroom 313 recorded 1508ppm. Classroom 313 experienced a higher average value at 1867ppm.

The cumulative relative frequency graphs show in percent how much time students spent in an environment below 1500ppm (Figure 16 for classroom 311, Figure 18 for classroom 312 and Figure 20 for classroom 313). These data show that classroom 311 had the worst environment, where students spent only 21% of their time in an environment below 1500ppm CO_2_. In classroom 312, students spent 52% of their time in an environment below 1500ppm CO_2_, and in classroom 313, students spent 50% of their time in an environment below 1500ppm CO_2_. Figure 17, Figure 19 and Figure 21 for classrooms 311, 312 and 313, respectively, show the measured concentrations over the course of the analyzed time periods and clearly indicate that better environments are achieved with more intensive ventilation.

Table 4 summarizes the statistical information of the data collected for the period 7:00 AM–3:00 PM.

## 5. Conclusions

The proposed and subsequently implemented monitoring system was designed to monitor air quality in interior rooms, mainly classrooms and laboratories. However, it can also be applied to other school premises, offices, or households.

The air quality monitoring system was deployed at two locations, the first at the Department of Cybernetics and Biomedical Engineering, VŠB-TUO (office EA341, laboratory EB305), and the second at the Gymnasium and Secondary School of Electrical Engineering and Computer Science, Frenštát pod Radhoštěm (classrooms 311, 312 and 313).

The measured values were analyzed for time periods the presence of people in the given room was expected. According to the data obtained in this manner, the portion of time people spent in an unsuitable environment could be determined.

The building, where rooms EA341 and EB305 are located, are equipped with air-conditioning units but without air recovery, therefore ventilating is still necessary. Classrooms 311, 312 and 313 are in an older building which has been renovated but is not equipped with air conditioning. Both buildings are therefore not equipped with air recovery.

The installed monitoring system allows measurement in intervals of one minute to several hours of CO_2_ concentration, temperature, and relative humidity of ambient air, and if required, atmospheric pressure. An interval of 5 min was selected as a sufficient period for measurements and data transmission.

The data obtained from the measurement devices were analyzed according to the limits stipulated by law. The measured values demonstrated that the CO_2_ concentration limits were sometimes exceeded. The graphs of other monitored quantities, i.e., temperature and relative humidity of the ambient air in individual monitored rooms, are a part of Appendix A of this article. These data show the effect of ventilation on the CO_2_ concentration. When the window is open, the room temperature decreases, and the relative humidity also decreases. Measured ambient air temperature values in the analyzed period did not exceed the tolerated band, see Table 1. Measured ambient air relative humidity values during the analyzed period did not exceed the tolerated band, but were below the lower limit of the tolerated band, see Table 1. The data were collected in winter (January 2019 at the Grammar School and Secondary School of Electrical Engineering and Computer Science, Frenštát pod Radhoštěm, and February 2019 for Department of Cybernetics and Biomedical Engineering, VŠB-TUO). These measured values are available on the university server enviro.vsb.cz [38] (login: sensors, password: sensors). Measured data from all monitored rooms at Grammar School and Secondary School of Electrical Engineering and Computer Science, Frenštát pod Radhoštěm in the period from 7 December 2018 to now are available on this website.

The graphs of the data showed that air quality in the rooms was significantly affected by how much they were ventilated. Greater ventilation is more effective at lowering CO_2_ concentrations to improve overall air quality. If rooms are ventilated regularly and CO_2_ concentration does not significantly exceed 1500ppm, the overall quality of the environment is satisfactory.

The paper dealt with the description of an extensive monitoring system for measuring air quality in rooms. This is the original design of a comprehensive monitoring system, which was first tested at the authors’ department and later installed at the Grammar School and Secondary School of Electrical Engineering and Computer Science, Frenštát pod Radhoštěm. Data from this monitoring system can be used to design an automated ventilation system. The duration and intensity of ventilation and number of people in the room played a significant role in this situation. An important consideration is that the CO_2_ concentration level can be indicated directly on the RMCD device, which allows ambient air quality in the monitored room to be immediately assessed. Another advantage of the described solution is acoustic signaling when the limit concentration of CO_2_ is exceeded at 5000ppm. Indication of CO_2_ concentration in the monitored room significantly affects the behavior of people in the monitored room. Based on the authors’ experience, it can be observed that by direct indication of CO_2_ concentration people in monitored rooms tended to open windows more often, as can be seen from the graphs available online enviro.vsb.cz [38] (login: sensors, password: sensors).

## Figures and Tables

**Table 1:** Limit values of measured quantities.

	Min. Value	Rec. Value	Max. Value
CO_2_ concentration (ppm)	-	800–1000	1500
Temperature (°C)	20 (except. 18)	20–24	28 (except. 30)
Relative humidity (%)	30	30–64	70

**Table 2:** Timetable for 15 January 2019 at the Grammar School and Secondary School of Electrical Engineering and Computer Science, Frenštát pod Radhoštěm.

Lesson	1	2	3	4
Time	8:00 AM–8:45 AM	8:55 AM–9:40 AM	9:50 AM–10:35 AM	10:55 AM–11:40 AM
311	6th class	6th class	2nd class	6th class
312	7th and 3rd class	7th and 3rd class	7th class	7th class
313	2nd class	2nd class	free	2nd
**Lesson**	**5**	**6**	**7**	
**Time**	**11:50 AM–12:35 PM**	**12:45 PM–13:30 PM**	**13:35 PM–14:20 PM**	
311	6th class	4th class	free	
312	2nd class	5th class	free	
313	2nd class	4th class	8th class	

**Table 3:** Summary of statistical information of the data collected on 18 of February 2019 at the Department of Cybernetics and Biomedical Engineering, VŠB-TUO.

Room Number	Mean (ppm)	Min (ppm)	Max (ppm)	Standard Deviation (ppm)
EB305 laboratory	916	434	2051	484
EA341 office	792	561	1088	150
**Room Number**	**Variation (ppm^2^)**	**Median (ppm)**	**under 1500 ppm (%)**	
EB305 laboratory	234,139	856	84	
EA341 office	22,515	787	100	

**Table 4:** Summary of statistical information of the data collected for 15 January 2019 at the Grammar School and Secondary School of Electrical Engineering and Computer Science, Frenštát pod Radhoštěm.

Room Number	Mean (ppm)	Min (ppm)	Max (ppm)	Standard Deviation (ppm)
311	1867	716	2719	537
312	1567	855	2402	385
313	1508	671	3263	635
**Room Number**	**Variation (ppm^2^)**	**median (ppm)**	**under 1500 ppm (%)**	
311	288,068	1941	21	
312	148,281	1484	52	
313	403,594	1506	50	

## Abbreviations

The following abbreviations are used in this manuscript:

BLE	Bluetooth Low Energy
CO_2_	Carbon Dioxide
DPA	Direct Peripheral Access - byte-oriented protocol used to control IQMESH network services
EA341	Name of the office at VŠB-TUO
EB305	Name of the laboratory at VŠB-TUO
IQMESH	A topology type in an IQRF^®^ wireless network
IQRF^®^	Trademark name of the wireless communication technology for the nodes and coordinator in the MESH topology network
JSON	JavaScript Object Notation—An open-standard file format or data interchange format that uses human-readable text to transmit data objects consisting of attribute-value pairs and array data types (or any other serializable value)
KNX	An open standard (see EN 50090, ISO/IEC 14543) for commercial and domestic building automation
LoRa	Long Range—A low-power wide-area network (LPWAN) technology working in license free sub-gigahertz band
MQTT	Message Queuing Telemetry Transport—machine-to-machine (M2M)/“Internet of Things” connectivity protocol
OTA	Over The Air—firmware changes over the wireless network
REST-API	Representational state transfer—Application Interface
RMCD	Room Measurement of Carbon Dioxide—A device for measuring CO_2_ concentration
MySQL	An open-source relational database management system
VŠB-TUO	Vysoká škola báňská—Tecnická univerzita Ostrava—name of the university
